# The Need for a Code of Conduct for Research Funders: Commentary on Values in University-Industry Collaborations: The Case of Academics Working at Universities of Technology

**DOI:** 10.1007/s11948-019-00145-9

**Published:** 2019-10-12

**Authors:** Bert van Wee

**Affiliations:** Delft University of Technology, Faculty Technology, Policy and Management, PO Box 5015, 2600 GA Delft, The Netherlands

**Keywords:** Code of conduct, Research, Research ethics, Research funders

## Abstract

In addition to a code of conduct for researchers, it is desirable to implement a code of conduct for funders of research. This is because researchers often behave unethically as a result of direct and/or indirect pressure from funders. The paper provides an expansion of the first proposal for such a code of conduct and includes several elements such as “policy relevant research should not be contracted and supervised by a client with an interest in the outcomes”, and “policy relevant research should always be examined by an independent institute”.

Codes of conduct and related literature on research ethics generally focus on researchers. This also applies to the article by Rafaela Hillerbrand and Claudia Werker (2019). Researchers indeed do have important responsibilities, and codes of conduct could be helpful to researchers in avoiding unethical behavior. As Hillerband and Werker argue, especially when universities and industry partners collaborate, this collaboration is prone to value conflicts. As they make clear, such conflicts do not only apply to research but also to education and job training.

An additional and complementary way to consider research ethics is to focus on the funders of research. Indeed, a code of conduct explicitly for research funders is needed (van Wee 2015). In this paper, the emphasis is on research, but the scope is broadened beyond industry to include other funders of research with an interest in the outcomes, especially policy makers.

A great deal of research in some areas, such as the spatial sciences and transportation research is aimed at being relevant to policy making, especially studies of the effects and implications of different, potential policy measures. This is particularly an issue for practice-oriented or applied research, but in some cases for academic research as well. Therefore, critically important is whether politicians, policy makers, interest groups and the public can rely on the results. It is not only very important that research be of high quality, but also that it is carried out and reported independently and without bias. To give a few examples: a government minister should impartially present information to members of parliament rather than in a manner designed to persuade them to accept her policy proposal; interest groups should be objectively informed about the environmental impacts of a potential new rail line; research studying the effects (aimed and unintended) of a new drug must be conducted by the researcher without being influenced by the funder of that research, a pharmaceutical company, because it has a financial interest in the research findings.

Surprisingly there is not much literature studying the impact of funders (and other clients) on research results, despite the fact that research ethics is a topic that has long been discussed. There is, however, literature on codes of conduct, also called ethical codes, which aim to avoid, or at least reduce misbehaviour by researchers. Such codes are generally formulated at the level of universities or unions of universities, for professional organizations in several areas like engineering or consultancy, or at the company level. An example of a code of conduct for academia is the code of the Dutch Association of Universities in the Netherlands (VSNU), which emphasizes honesty, scrupulousness, transparency, independence, and responsibility (VSNU 2018). In more general terms, codes of conduct express what is considered to be responsible behaviour on the part of the members of an organisation or community (Bullock and Panicker 2003; Resnik 2007; van de Poel 2009).

An important question is whether such codes are enough to improve the quality of research by increasing awareness about research ethics, and by incentivizing critical thinking among researchers and engineers. A study of the dilemmas of researchers in the Netherlands who conducted cost–benefit analyses (CBA) of transportation suggests that it is doubtful that codes of ethics for researchers are enough (van Wee and Molin 2012). Interviews with eight researchers in the CBA community were used to derive propositions that were then sent to a larger group of researchers. Respondents (N = 28) scored the extent of their (dis)agreement with the propositions. Results of the study reveal that several dilemmas result from the behaviour of representatives of the funders. That is, often a ministry, province, or local municipality funds research and the officials of these government entities sometimes try to influence the research results in which they have an interest (van Wee and Molin 2012). Similarly, Bent Flyvbjerg and colleagues (2003) concluded that funders very likely influence researchers because large infrastructure projects tend to incur cost overruns across the world, not only due to an optimism bias (estimates that are too low as a result of too much optimism about the costs), but also due to “strategic behaviour” (also called “manipulation” or “lying”) by those people or institutions with an interest in a positive decision to build. This is likely to be an international, multidisciplinary problem.

It would seem that, to the extent that funders of research aim to influence research results, it is probably not enough to have only a code of conduct for researchers. A code of conduct for the funders of research is also necessary. The general aim of such a code should be that the client requests research that is “fit for purpose”. In other words, the report of research findings should be a useful tool to inform different users of the research in an unbiased and adequately informative way. The quality of the research should be sufficiently sound to support a policy decision based on the results: “A practical rule of thumb could be that the quality of public decision making is higher, if the decision makers make the choice they would have made: (1) if they would have all (from their perspective) potentially relevant choice options available, (2) if they were fully informed, and (3) if they were able to evaluate different choice options.” (van Wee 2011, p. 20).

The Textbox below provides some initial ideas. More insights into current practice and the dilemmas for researchers are needed to create an actual code of ethics for funders of research.

**Table:** 

Textbox: Options for a code of ethics for clients of research (based on van Wee 2015)
A general rule, and probably the most important rule, is that policy relevant research should not be contracted and supervised by a client with an interest in the outcomes. An option is to let independent institutes like the National Science Foundations select the research institute and supervise the research
Certainly if the above rule was not implemented, but maybe even if it is, another rule could be that policy relevant research should always be examined by an independent institute. In the research by van Wee and Molin (2012), 23 out of 28 respondents (strongly) agreed with the proposition ‘Asking for a second opinion leads to a higher quality of [cost–benefit analysis]’. The interviews preceding the propositions-based questionnaire revealed that, often communicating to the client that, a second opinion would be carried out was a sufficient reason, for the client to stop trying to influence the results
Researchers that are put under pressure to change methodology or results have the right, or even the duty, to make this public. This could also prevent clients from manipulating, or trying to manipulate, researchers
Clients cannot prescribe the models, other tools or scenarios that would result in a not objective/impartial estimation of the effects of policy options. In the case of policy measures with long-term impacts (like infrastructure projects), using at least two contrasting scenarios for economic and other societal developments could be made obligatory, in order to make uncertainty in the outcomes explicit, at least to some extent
Researchers have the right to publish their results in journals and communicate these to the media

Will such a code of conduct for research funders be enough to avoid manipulation by funders of research? Maybe not. Some researchers have argued that a more systems-based approach is needed. Such an approach would focus on the system of research as a whole, including both researchers and the funders of research, and perhaps even more actors like the users of the research (te Brömmelstroet and Korff de Gidts 2014). While a systems approach might be the better option, it is likely to be a long time off, if related experience is any indicator. In the year 2000 a guideline for national, large-scale transport infrastructure projects was implemented in the Netherlands (Eijgenraam et al. 2000). The guideline prescribed a CBA and how to conduct it. However, it was 13 years later before a cross-ministry guideline for the ex ante evaluation of candidate policy options was implemented (Romijn and Renes 2013). This guideline recommends the use of a CBA in many (but not all) cases, and the procedures required to carry it out. It emphasizes that the use of a CBA can structure the process of policy preparation. This 13-year process suggests that it might, therefore, be better to start discussing and implementing a code of conduct for research funders first. In the end, codes of conduct for researchers as well as for funders of research could evolve into a more systems-based approach.

What would be the effects of a guideline for funders of research? It is difficult to give an adequate answer to this question, and indeed, this is an interesting and relevant research area in itself. Research questions could focus on similarities and differences in dilemmas for different categories of researchers (academic research, practice-oriented research), different research and policy areas (such as transport, spatial planning, housing, health, education), different (clusters of) countries, and different institutional settings. Research could also study the effects of ethical codes on researchers.
